# Machine learning spectroscopy to advance computation and analysis

**DOI:** 10.1039/d5sc05628d

**Published:** 2025-11-06

**Authors:** Julia Westermayr, P. Marquetand

**Affiliations:** a Wilhelm-Ostwald-Institut für Physikalische und Theoretische Chemie, Universität Leipzig Linnéstraße 2 04103 Leipzig Germany julia.westermayr@uni-leipzig.de; b Center for Scalable Data Analytics and Artificial Intelligence (ScaDS.AI) Dresden/Leipzig Germany; c Quastify GmbH Carl-Friedrich-Gauss-Ring 5 69124 Heidelberg Germany

## Abstract

Spectroscopy, the exploration of matter through its interaction with electromagnetic radiation, is relevant in many diverse research fields, such as biology, materials science, medicine, and chemistry, and enables the qualitative and quantitative characterization of samples. Machine learning has revolutionized spectroscopy by enabling computationally efficient predictions of electronic properties, expanding libraries of synthetic data, and facilitating high-throughput screening. While machine learning has strengthened theoretical computational spectroscopy, its potential in processing experimental data has yet to be adequately explored. At the same time, automating structure and composition predictions from spectra remains a formidable challenge that requires theoretical simulations and expert knowledge. This review addresses the synergy between machine learning and spectroscopy, covering various techniques including optical, X-ray, nuclear magnetic resonance, and mass spectrometry. It outlines the fundamentals of machine learning, summarizes the techniques, and previews future developments to fully exploit the potential of machine learning and advance the field.

## Abstract 2

Spectroscopy enables studying matter *via* its interaction with electromagnetic radiation, supporting analysis, with machine learning further advancing its capabilities.

## Graphical abstract

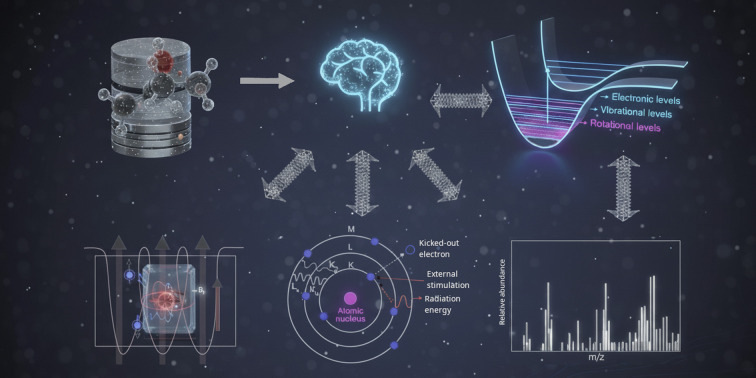

## Introduction

Spectroscopy, the study of matter through the interaction with electromagnetic radiation, dates back to Isaac Newton's experiments on prisms that split light into its constituent wavelengths in the 1660s. Since then, it has evolved into a versatile set of techniques that are crucial to various fields, including biology, material sciences, medicine, and chemistry. Spectroscopy is valuable for characterizing samples qualitatively and quantitatively, and time-resolved experiments can elucidate dynamic changes in response to perturbations. Nevertheless, achieving the automated prediction of a sample's structure and composition based on a provided spectrum continues to be a formidable task.

The growing complexity of experiments has further complicated the comprehension of structures, compositions, and mechanisms within intricate samples. Therefore, theoretical simulations are often needed to assist in interpretation, but they are limited by the high computational efforts required for underlying quantum chemical calculations. In addition, standard approaches that aim to predict structures based on a spectrum often rely on search engines and spectral libraries, which can miss compounds not present in screened libraries. Expert knowledge and chemical intuition are often necessary, especially for complex sample characterization with mixed chemicals. As a consequence, studying molecules and materials in realistic environments and on experimentally relevant time scales remains challenging.^1^

The use of machine learning (ML) has greatly advanced spectroscopy, revolutionizing existing computational theoretical techniques by enabling computationally efficient predictions of electronic properties. This breakthrough has not only facilitated advancements in computational high-throughput screening but also enabled the study of larger scales over longer periods. Specifically, ML algorithms have increased the efficiency of predicting spectra based on a given structure, resulting in the enhancement and expansion of libraries with synthetic data. While this advance has made theoretical computational spectroscopy an effective tool for supporting and complementing experimental results, the full potential of ML in the field of spectroscopy, especially in the context of experimental data, has yet to be exploited. This review aims to explore spectroscopy through the lens of ML, examining the challenges and prospects for enhancing various spectroscopic methods with ML, from both, an experimental and theoretical point of view.

The review starts with a brief overview on ML, summarizes the different ML techniques and models used in spectroscopy and concludes with a discussion on open challenges and possible avenues towards further enhancing spectroscopy with ML. The spectroscopic techniques covered in this review are optical spectroscopy using light in the ultraviolet (UV), visible (vis) and infrared (IR) region, X-ray spectroscopy, nuclear magnetic resonance spectroscopy (NMR), and mass spectrometry (MS). We will not cover surface analysis tools like atomic force microscopy (AFM) and transmission electron microscopy (TEM) and refer the reader to excellent reviews on this topic.^1,2^

## Machine learning overview

ML has emerged as a state-of-the-art method for predicting electronic properties in chemistry, accelerating molecular dynamics simulations and spectra computations. However, ML for experimental data is still in its infancy and faces several challenges. To advance experimental and theoretical spectroscopy, we will outline the basic ML concepts.

ML techniques can learn complex relationships within massive amounts of data that are difficult for humans to interpret visually. Three main types of algorithms exist: supervised learning, unsupervised learning, and reinforcement learning. In contrast to classical physical models, which are based on assumptions made by researchers, ML models can learn, at least in principle, any arbitrary function, *f*, that maps an input space *X* to a query space *Y*: *f*:*X* → *Y*. This capability allows for computational efficiency because, once trained on data from expensive *ab initio* simulations, ML models enable rapid inference of properties like electronic energies or spectra, often orders of magnitude faster than traditional quantum-chemical methods.

### Supervised learning

Training, *i.e.*, learning, is achieved by minimizing a so-called loss function, *L*. Therefore, randomly initialized model parameters are optimized during training. In supervised learning, the loss function requires that the target properties, *y* ∈ *Y*, are known. The loss function to be optimized, *L*(*f*(*x*), *y*), can thus be formulated by computing the error between the predicted values *f*(*x*) and the query values, *y*, in the training set: *L*_*n*_ = *f*(*x*) − *y*^*n*^, with *n* referring to the norm of the loss function. *L*_1_ and *L*_2_ are among the most frequently used loss functions.

**Fig. 1:**
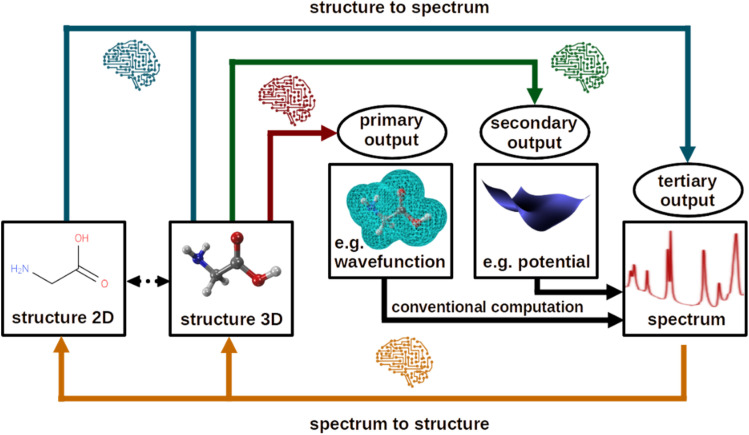
Use of machine learning for computational spectroscopy. The use cases can be divided into (i) from structure to spectrum, and (ii) from spectrum to structure. The former can be subdivided into (a) learning primary outputs of quantum chemistry calculations (*e.g.*, wavefunctions) and using conventional techniques to compute spectra, (b) learning secondary outputs (*e.g.*, potential energy surfaces), and (c) learning spectra directly, which can be seen as tertiary outputs.

Most supervised ML models that have been developed in the last couple of years are based on theoretically computed quantum chemical data to learn either primary, secondary, or tertiary outputs,^4^ see Fig. 1. These types of models refer to regression models. Learning the primary output of a quantum chemical calculation (red arrow in Fig. 1), which could be, for instance, the electronic wavefunction – from which any property one wishes to know could be calculated – is probably the most powerful way but requires a 3-dimensional structure. At the same time, it is also the most complex task to be achieved as the electronic wavefunction is high-dimensional and dependent on all electrons of a molecule or material. Hence, this task is still an unsolved challenge, especially when dealing with multiple molecular systems in one ML model, multiple electronic states, or materials that need consideration of an infinite number of electrons. A recent perspective summarizes approaches developed to advance the search for a ML-enhanced solution of the Schrödinger equation.^5^

Most ML models applied in spectroscopy predict the secondary output of a quantum chemical calculation (green arrow in Fig. 1). A secondary output is a property that can be computed from the Schrödinger equation, such as the electronic energy, dipole moment vectors, or couplings, of which tertiary outputs (blue arrow in Fig. 1), such as the oscillator strength or a spectrum, can be computed. Knowing the secondary output is beneficial compared to learning the tertiary output directly as usually more physical information is available. For instance, by learning electronically excited states and transition dipole moment vectors, absorption spectra can be computed *via* convolution and information about the contribution of different electronic states to different peaks in a spectrum can be obtained. When learning spectra directly, which could be done by distributing a spectrum into a certain number of data points, this information is lost. When using experimental data, the only viable way is most often the learning of tertiary outputs. Again, the task of learning secondary outputs requires 3-dimensional structures for accurate predictions as secondary properties exhibit a stronger dependence on precise 3D geometry due to their direct ties to electronic structure variations across conformations, making 2D representations like SMILES strings insufficient for accurate prediction without significant loss in fidelity. For instance, in IR spectroscopy, learning secondary outputs such as electric dipole moments from molecular dynamics trajectories demands robust handling of conformational ensembles, whereas direct prediction of the tertiary spectrum integrates these variations implicitly, allowing viable approximations from 2D inputs that focus on overall composition rather than atomic positions.

Learning experimental data compared to theoretical data is powerful as ML models could directly be coupled with experiment and often, the holy grail of theoretical simulations is to achieve experimental accuracy. However, the learning of experimental data is still in its infancy. Reasons include limited data available and the inconsistency in generated data that stem for instance from human constitution or different experimental setups and protocols used within different researchers. Another disadvantage is that additional insights into electronic structure and interpretability are lost. Systematic theoretical calculations can significantly reduce human effort.

Besides regression models, classification tasks fall under the category of supervised learning, which can be used to find patterns and groups in data. In chemistry, classification tasks can be, for instance, to select an appropriate quantum chemistry method^3–5^ or to classify biological systems like enzymes.^6^

As one usually deals with finite training sets, meaning that many possible solutions to map inputs, *x* ∈ *X* (where *x* is one input from the set of possible inputs *X*), to outputs, *y* ∈ *Y*, exist. Looking only at training instances in supervised learning, it can thus happen that functions that are overly complex are used to fit simpler functional relationships, leading to generally bad generalization. This event is known as overfitting and can be avoided by regularization, which adds a penalty to complex solutions. Further, to obtain a good fit, it is important that the training set is sufficiently large and covers the chemical space of interest comprehensively. This requirement makes the application of ML to experimental data extremely difficult, as experiments are often costly and time-consuming, hence limiting the amount of data that can be produced. In addition, experimental data can depend on factors that are hardly controllable when executed by humans, such as fluctuations when weighing samples or errors due to varying human constitution. The automation and miniaturization of chemical processes thus offers great promise for high-throughput experiments and consistent data generation.

### Unsupervised learning

In contrast, unsupervised learning is concerned with finding patterns in data without access to target properties, *y*. Unsupervised learning techniques used in chemistry include dimensionality reduction, such as principal component analysis, or clustering, which are mainly used to post-process and analyze data,^10,11^ respectively, or generative models that can be used to learn from a data distribution to generate data similar to it, often applied in molecular design studies.^7–9^

### Reinforcement learning

Besides unsupervised and supervised learning, reinforcement learning exists, which is learning from interaction with an environment and corresponding rewards/punishments. Reinforcement learning is what humans do. For instance, when we play a game like chess, we learn how to strategically adapt our actions to win based on our experience while playing.^13^ In reinforcement learning, similarly, an agent takes actions on an environment in a specific state. The action is defined by a probabilistic policy and is adapted by consideration of a reward, *i.e*., the immediate outcome of an action, and a value function, which is the long-term reward. As an example, playing chess is considered here. In chess, immediate rewards are gained by making moves that directly benefit the current position, while long-term rewards are achieved by making moves that contribute to winning the game overall, which may involve sacrificing immediate gains for strategic advantage. In this way, the agent can learn to adapt actions such that not only immediate actions, but also long-term rewards are maximized, enabling exploration in addition to exploitation of successful actions. While reinforcement learning usually ends up with lots of data, it has the advantage of allowing for exploration starting with little data. As a drawback, the model might explore inefficiently and can profit from restrictions or policies that guide initial exploration.^13^ Examples of reinforcement learning models are *e.g.*, AlphaGoZero, which has learned the strategy game Go *via* self-playing and has bet the world-best player. Examples of reinforcement learning in chemistry are sparse but have so far been used for transition state searches,^14,15^ identification of retrosynthetic pathways,^16^ and molecular design.^17^

### Molecular descriptors

One consideration that has to be made when applying ML to chemistry and spectroscopy is that representing a molecular geometry by *xyz* coordinates is usually disadvantageous. Rather, the input should be transformed into a rotationally and translationally invariant descriptor in order to incorporate physical principles and get by with small training set sizes. In general, local and global descriptors can be distinguished. Global descriptors are for instance the matrix of inverse distances, which cover the whole molecule and become larger the larger a molecule. As a result, the representation of large systems, like proteins or nanomaterials, is inefficient using global descriptors. The Coulomb matrix, for instance, scales with the number of atoms squared. Local descriptors offer a solution by representing atoms in their chemical and structural environment within a cutoff region that is seen by an atom. The cutoff is often a critical parameter as a too large cutoff leads to large descriptors, hence to inefficient training, but a too small cutoff can result in missing long-range effects. Models like Behler's fourth generation neural network potentials^10,11^ or external long-range dispersion calculators based on partial atomic charges have been developed that allow to account for long-range interactions implicitly or explicitly,^20^ respectively. The generation of a representation that is rotationally and translationally invariant typically means that we can go from a molecular structure to a prediction, but not the other way around, *i.e.*, the 3d molecular structure cannot simply be reproduced when given an output property. This has a direct consequence on the application of ML to spectroscopy, where an important task is the search and reconstruction of 3d structures of molecules and materials from a given spectrum (going from right to left in Fig. 1). For a more detailed overview of the representation of molecules and materials for ML, we kindly refer the reader to ref. 21.

## Spectroscopy overview

**Fig. 2:**
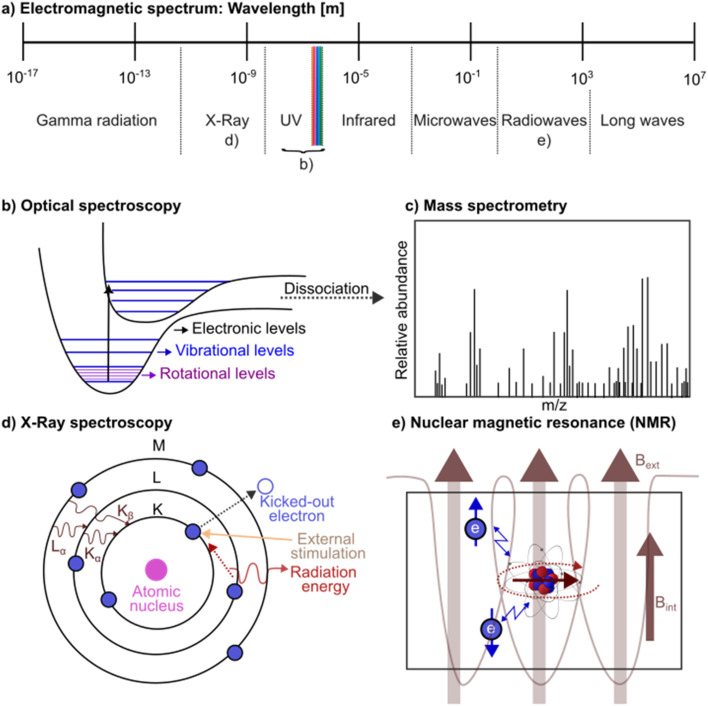
Overview of spectroscopic techniques covered in this review. (a) Electromagnetic spectrum. (b) Potential energy surfaces that represent electronic energy levels. Each electronic energy level (black) is divided into vibrational energy levels (blue) that are themselves divided into rotational energy levels (purple). The potential energy curves are dissociative. Dissociated systems could be detected in a (c) mass spectrum that shows the relative abundance of fragments with a certain mass to charge (*m*/*z*) ratio. From a theoretical point of view, molecular dynamics simulations can be conducted that then help interpret the mass spectrum. (d) X-ray spectroscopy using the Siegbahn notation. K, L, and M denote the electron energy levels, with K being the closest to the nucleus. *α* and *β* represent transition sizes. M to L or L to K transitions are labeled as L_*α*_ or K_*α*_, while M to K transitions are referred to as K_*β*_. (e) Nuclear magnetic resonance, where nuclei interactions rather than those of electrons are measured using a magnetic field.

Spectroscopy is concerned with the study of the interaction of matter with electromagnetic radiation and can be classified in a number of ways, such as by the type of material studied, the nature of the interaction, *i.e.*, absorption, scattering, or emission, or by the type of detection. Analysis involves how effects change with varying energy of radiation. In this review, we will classify techniques by the wavelength of radiation used to excite a molecular system or material. Fig. 2a shows the electromagnetic spectrum and corresponding spectroscopic techniques that use different spectral regions. A spectrum is often recorded by measuring changes in the intensity, frequency, polarization, or other properties of radiation or induced signals arising from interactions such as absorption, emission, scattering, reflection, or free induction decay when the radiation interacts with a sample. Samples can be identified as each chemical system has a unique spectral fingerprint that can be analyzed and matched against a database.

## Optical spectroscopy

Some of the earliest ML works on spectroscopy are applied to a set of techniques summarized under the term optical spectroscopy (Fig. 2b). Optical spectroscopy involves transitions between rotational, vibrational, or electronic energy levels that occur *via* absorption or emission of light in the range of microwaves, infrared, or UV/vis light, respectively. In general, a quantum chemical system can only take certain discrete energy values, which are referred to energy levels. Energy levels can be separated into electronic energy (*E*_elec_) levels, which are ground and excited electronic states involving electron transitions (shown in black in Fig. 2b), vibrational energy (*E*_vib_) levels associated with oscillatory motion of atoms or groups of atoms in a molecule (shown in blue in Fig. 2b), and rotational energy (*E*_rot_) levels, the latter involving a change in the angular momentum of a molecule (shown in purple in Fig. 2b). The total energy of a molecule (including translational kinetic energy) can then be written as:*E* = *E*_rot_ + *E*_vib_ + *E*_elec_.

Excellent reviews on deep learning for Raman spectroscopy,^22^ near-IR spectroscopy,^23^ vibrational spectroscopy in general,^24^ and data analysis of optical spectra^25^ can be found in ref. 12–15.

### From structure to spectra

**Fig. 3:**
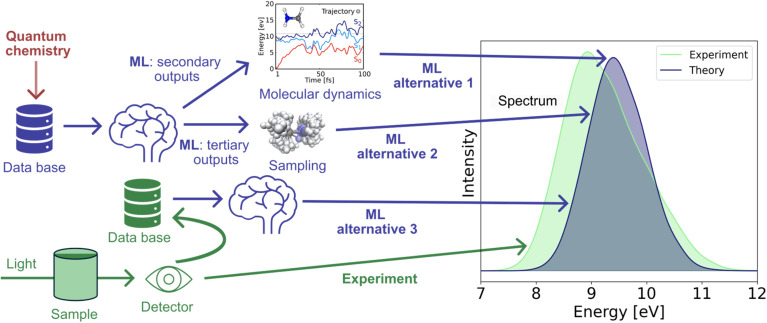
Different ways to generate absorption spectra. With quantum chemistry, electronic properties like energies and forces (secondary outputs) or transition probabilities between quantum levels like oscillator strengths (tertiary outputs) of a molecule can be computed. Machine learning can be used to predict these properties for many conformations. The latter can be obtained either *via* molecular dynamics and then calculating an autocorrelation function to get a spectrum (ML alternative 1) or *via* sampling and subsequent convolution (ML alternative 2). Another alternative is to collect experimental spectra of many different molecules and learn from this data to predict spectra of unseen compounds (ML alternative 3).

Optical spectra can be computed either statically by taking a pre-determined structure of a molecular system as input or dynamically from a set of structures obtained from a time-dependent simulation. This is exemplified in Fig. 3, top right. While the former is powerful for high-throughput screening, the latter provides a more accurate picture of the spectroscopic fingerprint of a system and goes beyond the assumption of a simple harmonic oscillator, including finite temperature effects.

To obtain a UV/vis absorption spectrum, information about the excitation energies between the ground state, *E*_0_, and an excited state, *E*_j_, as well as the oscillator strength, *f*^Osc.^_0j_, obtained from the corresponding transition dipole moment, *μ*_0j_, is needed:
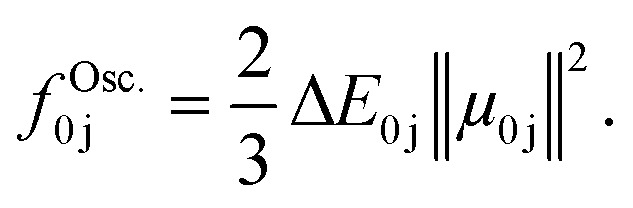


The oscillator strength provides information about the intensity of a peak at the position of the excitation energy, Δ*E*_0j_. A spectrum can be obtained *via* convolution with a line shape function, often a Gaussian or Lorentzian function, using a pre-defined width, which is dependent on the system studied and the number of molecules sampled. This line shape function mimics line broadening effects because of *e.g.*, molecular motion. The latter is better represented when sampling structures *via* molecular dynamics or a Wigner distribution. Note that in case of molecules exhibiting different conformers, such as it is the case with amino acids or peptides, it is important to sample the different conformers and weighing their contributions accordingly, for instance *via* a Boltzmann distribution.^26^

The computation of IR spectra can be done *via* the Fourier transform of the time autocorrelation function of the dipole moments with the intensity, *I*_IR_, depending on the time derivative of the molecular dipole moments, *μ̇*_j_, of a given state, j:
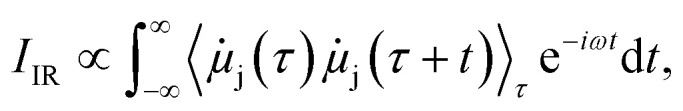
where *ω* denotes the vibrational frequency and *τ* a time lag. In contrast, Raman spectroscopy relies on scattering events and depends on the polarizability tensor, of which derivatives with respect to normal modes are computed in case of static calculations.^13,14^ Instead of the dipole moment, the autocorrelation function can be formed of the polarizability tensor for Raman spectra in case of dynamics simulations. Both, Raman and IR spectra are powerful to obtain information on chemical bonding and structural arrangement in samples.

One of the first ML studies applied to spectroscopy was reported in 1993, when Affolter *et al.* trained neural networks on 306 IR spectra of organic compounds. In their approach, tertiary outputs, meaning the spectra, were discretized into 1801 data points each between 4000 and 400 cm^−1^ outputs, that were then learned based on 5 structural descriptors.^125^ Both concepts are exemplified in the bottom of Fig. 3. Later in 2015, the first studies dealt with the prediction of UV/vis absorption spectra by learning secondary outputs, which are the oscillator strengths and excitation energies at time dependent density functional theory (TDDFT) level of theory. To this end, the QM9 data set (a benchmark database for small molecules) was used in combination with kernel ridge regression models. The quality of the spectra could further be improved by a Δ-ML model trained on the difference between TDDFT and CCSD properties.^29^ For learning oscillator strengths, which are scalar values, conventional ML models can be used for static (*i.e.*, single point quantum chemical calculations of sampled structures that are obtained for instance *via* Wigner sampling) as well as dynamic (*i.e.*, *via* autocorrelation functions of electronic properties obtained from molecular dynamics simulations) absorption spectra calculations.

In the context of IR And Raman spectroscopy, Ren *et al.*^16^ trained two separate neural networks on vibrational frequencies and IR and Raman intensities of molecules in the QM9 data set, respectively. In the condensed phase, data is often limited, hence Kananenka proposed^30^ an approach for improving the accuracy of the prediction of OH-stretch frequencies and dipole derivatives of liquid water. Therefore, Gaussian process regression models were used to generate a data set *via* molecular dynamics that was then used to train artificial neural networks.^17^ When dealing with molecules on surfaces, surface-enhanced Raman spectroscopy can capture electronic-vibrational fingerprints. To advance this technology, Hu *et al.* used random forest models to predict vibrational frequencies and Raman intensities of *trans*-1,2-bis(4-pyridyl)ethylene adsorbed on a gold surface.^18^ Park and co-workers^19^ have developed a deep graph convolutional neural network to predict seven properties as single values of organic compounds to characterize optical properties. In addition, the authors included environmental effects to study influences of environments on optical properties. The method has been applied for screening to design a blue emitter with target optical and photophysical properties. Learning these properties can be applied, at least in principle, to any type of systems and can be seen as a very general and versatile approach of using ML for optical spectroscopy. The accuracy of the predictions typically follows the one of the training data. However, limitations might arise when trying to interpret which electronic state contributes to which peak in a spectrum. Here, an attribution is often impossible. Therefore, it can be beneficial to learn energies and dipole moments instead.

Compared to learning single values like energies or oscillator strengths, learning vectorial properties like dipole moment vectors requires additional considerations. Either equivariant representations are required to accurately predict vectorial or tensorial properties with ML, which has been shown to be accurate for the prediction of spectra in polarizable atom interaction neural network (PaiNN),^20^ or physical relations need to be incorporated into the ML model for accurate learning. The latter approach has been conducted *via* the charge model, developed by Gastegger *et al.*,^21^ who used the model to predict IR spectra from molecular dynamics simulations. The charge model makes use of the relation of the dipole moment vectors with the atomic partial charges, *q*_a,j_, of atom a, and the distance of each atom to the center of mass of the molecule with *N*_a_ atoms, *r*^COM^_a_:
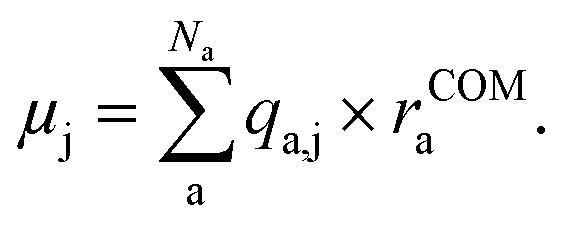


By learning the dipole moment vector *via* inferring the atomic partial charges and using the above relation, vectors can be predicted accurately and in addition, information on atomic partial charges based on the reference method can be obtained.^21,22^ Worth mentioning is that this model fails to capture out-of-plane transition dipole moment vectors when planar molecules are described. This problem has been solved by Zhang *et al.*,^23^ who built the dipole moment vector *via* the addition of three independently predicted vectors, two of which, *μ*_T_^1^ and *μ*_T_^2^, should be non-identical and are obtained by the above equation. The third one is obtained *via*:
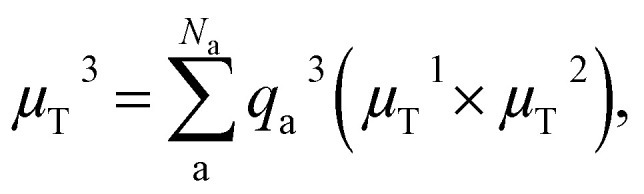
guaranteeing that the third vector is perpendicular to the plane spanned by the first two vectors. This approach has been used for the prediction of IR spectra and vibrational as well as electronic spectra of *N*-methylacetamid and proteins and is reviewed in detail in ref. 24.

Another challenge that arises when modeling properties that arise between two electronic states, like transition dipole moment vectors, is due to the arbitrary phase of the wave function, which leads to inconsistent signs, impeding training of conventional ML models. Therefore, data has to be corrected with respect to this inconsistent phase or a phase-free training algorithm has to be applied that allows for the learning of transition dipole moment vectors.^25^ Different approaches for phase correction exist and are reviewed in ref. 26. With the phase free training algorithm, the absorption spectra of small molecules could be predicted and transferability across chemical compound space has been shown.^22^

ML is especially powerful when it comes to transferring knowledge from small building blocks to large systems, advancing the prediction of extended systems, such as molecular crystals or proteins, of which data are hardly accessible.^23,28^ Zhang *et al.*, for instance, learned amino acid residues and peptide bonds to predict a Frenkel exciton Hamiltonian with which UV/vis spectra of proteins could be computed with high accuracy.^23^ A similar approach that cuts proteins into peptide bonds and amino acids that are independently modeled with ML was conducted by Zhao *et al.* to predict protein circular dichroism spectra.^29^ Circular dichroism spectra are based on electronic transitions and are highly sensitive to structural changes in a protein's structure. They were computed from electric and magnetic transition dipole moments of peptide bonds predicted with ML using embedded density descriptors. Besides IR and absorption spectra, Raman spectra were modelled by Raimbault *et al.* using symmetry-adapted Gaussian process regression. Therefore, static polarizability and dielectric susceptibility of molecular crystals were predicted during molecular dynamics to obtain anharmonic vibrational spectra.^30^ Another powerful approach to achieve transferability is the application of foundational models, which have been demonstrated for molecules and materials for a variety of applications,^31–33^ such as molecular dynamics of nanoparticles using the message-passing atomic cluster expansion model,^34^ MACE-off,^35^ oscillator strength predictions^36^ and have recently been adapted for excited states using X-MACE.^37^ In general, equivariance has shown to improve transferability also in the excited states, as, *e.g.*, shown with SPaiNN,^38^ the equivariant ML-based photodynamics approach coupling PaiNN^39^ and SHARC.^40^ While still in early stages, these models have shown to advance the prediction of spectra^36^ and molecular dynamics for subsequent spectra predictions. Very recently, a first attempt for a foundational model for IR spectroscopy, namely MACE4IR, has been developed showing promising results for complementing experiment without or minimal additional data.^41^

The above-mentioned studies predict molecules and materials in the gas phase. However, most chemistry happens in environments or solution. To take environmental effects into account, different approaches are possible with the simplest approach being the inclusion of environmental effects *via* implicit solvation models. These can be incorporated during quantum chemical calculations; hence no further ML adaptions are needed when only one solvent is treated. Another way would be to treat the environment explicitly, for instance, *via* a quantum mechanics/molecular mechanics approach, where the environment is described classically, influencing the molecule under investigation, the latter being treated quantum chemically. One way to include environmental effects into ML has been developed by Gastegger *et al.* in FieldSchNet.^42^ This neural network approach can model molecules in different solutions by adding an additional term to the descriptor, which is the external field, *ε*_ext._(*R*_a_) at atom a. This property is based on the atomic partial charges of surrounding atoms *N*_k_, including the environment:
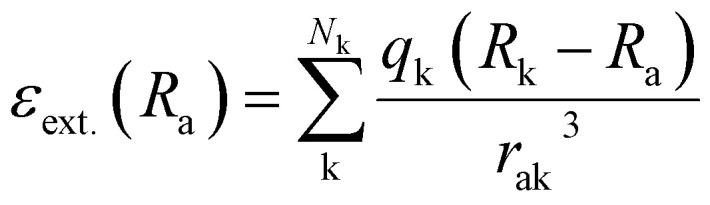


FieldSchNet has been applied for the prediction of IR, Raman, and NMR spectra of molecules and reactions. FieldSchNet is very powerful to study molecules in solution and is very accurate to do so. In fact, it allows to assess the influence of different environments on the spectrum of a molecule under investigation. However, the method requires significantly more training data due to the sampling of the environment for a single conformation of a system of interest, making the method computationally more expensive. Also in this case, the difference in accuracy between experiment and quantum chemical calculations used as training data is usually much larger than the difference between these quantum chemical calculations and the ML predictions. Recently, FieldSchNet has been adapted for excited-state simulations,^43^ allowing for ML-driven simulations of nonadiabatic molecular dynamics of molecules in environments, thus enabling the prediction of excited-state spectra. In addition, MACE and X-MACE have been extended using long-range blocks in the message-passing framework *via* the multipole expansion to simulate molecules in environments.^44^ Importantly, data efficiency could be improved in these models by transferring knowledge from ground-state foundational MACE models.

### From spectra to structure

While ML models have transformed the prediction of spectra, one might be interested in accomplishing the inverse, which is the prediction of a molecular structure or composition from a given spectrum. To date, no method exists that can achieve the goal of taking an electronic property, spectrum or dynamics result and predicting the corresponding structure(s). The probably most severe challenges to overcome in this respect are related to the fact that many spectra comprise an overlay of different conformers of a molecular system and that ML models cannot simply learn from *xyz*-coordinates but rely on a physics-based representation. While a unique mapping from a structure to such descriptors is relatively easy, the reconstruction of the three-dimensional structure is often not possible and alternative approaches that make use of existing libraries have been proposed.

First attempts into this direction have already been made over 20 years ago by Gasteiger and co-workers to obtain the conformation of molecules based on vibrational spectra.^45^ Therefore, the authors used counter-propagation neural networks that learned the relation between molecular radial distribution functions and IR spectra and could predict radial distribution functions from spectra. These functions have then been compared to functions of spectra in an existing data base of known systems.^46,47^ Guesses of structures that might result in the sought IR spectrum can be obtained, but the method is restricted by existing spectral libraries and conformations not present in the data set are missed. What is more is that in many situations, not only one, but a set of conformations is measured in experiments, which cannot be captured with this approach.

Another approach to assist structure identification from rotational spectra has been proposed by McCarthy *et al.*^48^ In rotational spectroscopy, rotational constants that are inversely proportional to moments of inertia, are conventionally used to identify structures by comparing experimentally obtained rotational constants with those obtained from quantum chemical calculations of query molecules. To assist structure identification, the authors have developed a set of neural networks, where the first one predicts Coulomb matrix eigenspectral from rotational constants, which is learned by three other neural networks to predict stoichiometries, SMILES strings, and most likely functional groups of the query molecule.

The prediction of functional groups and identification of mixtures based on Fourier-transform IR and MS spectra has been conducted by Fine *et al.*,^49^ who used an autoencoder to process spectral information and provides inputs for neural networks. Structural recognition of chemical groups from IR and Raman spectra of molecules with <10 heavy atoms has also been pursued by Ren *et al.*^16^ using long short-term memory networks. Liu *et al.*^50^ have advanced the identification of chemical species from Raman spectra obtained from the RRUFF mineral spectral database using convolutional neural networks.

The characterization of adsorption sites of complex interfaces has been conducted by Lansford *et al.*,^51^ who applied physics-inspired models that are trained on quantum chemistry data to predict synthetic IR spectra. These synthetic spectra were then used as inputs to two additional neural networks that learned the binding-type and general coordination number of an adsorbate *via* probability distribution functions to allow for structure elucidation. The latter models could be applied to both, experimental and synthetic data, further enabling uncertainty quantification.

### Spectra analysis and processing

Already in the 1990s least squares regression and neural networks have been used for data analysis. Visser and co-workers have tested these methods for pattern recognition in IR spectra and compared the results to those obtained from expert interpretation. The authors concluded that band shapes and patterns were better recognized by expert spectroscopists rather than by neural networks or least squares regression, but due to limited data, further investigations were required to support their results.^52,53^ Very recently, Dral *et al.*^54^ have applied Shapley values, a concept derived from game theory,^55^ to provide physically meaningful information about the impact of an input feature on experimentally curated two-photo absorption spectra. The authors found that by analysis of 900 molecules only a few features, such as the conjugation length, are important for the two-photon absorption magnitude. Finally, Guo *et al.* developed a protocol to standardize chemometric analysis of Raman spectra from data preprocessing to data learning to extract information in Raman spectra that arise due to small differences in spectra of related samples.^56^

Not only spectra analysis, but also data processing has been facilitated with ML. This is especially important as molecular peaks in spectra are often distorted by optical influences or entangled with background noise, hindering data interpretation. To overcome this issue, Guo *et al.*^57^ developed a 1-dimensional U-shape convolutional neural network to remove artefacts present in IR spectra. Training and test data were generated theoretically using Mie theory and was successfully applied to experimental spectra of poly(methylmethacrylate). An overview and review of analysis in Raman spectroscopy can be found in ref. 58.

### Outlook and perspective

While still in early stages, we believe that foundational models, especially for accelerating molecular dynamics simulations in both the ground and excited states, will dramatically advance the simulation of optical spectra. Foundational models like X-MACE,^37^ MACE-OFF,^35^ or MACE4IR(40) will enable real-time prediction of UV-vis, IR, and Raman spectra across diverse chemical spaces, facilitating high-throughput virtual screening of materials and pharmaceuticals. Physics-informed neural networks will address current limitations in environmental effects modeling, allowing accurate prediction of spectra in complex solvents and biological matrices without extensive retraining. In addition, interpretable and explainable AI has the potential to advance understanding of spectral–structure relationships, moving beyond black-box predictions to provide mechanistic insights into electronic transitions and vibrational design. This will enable discovery of new spectroscopic rules and improve confidence in ML-assisted structure elucidation.

## X-ray spectroscopy

While optical spectroscopy is non-invasive, higher energy radiation such as X-ray radiation or high-UV radiation can be used to remove electrons from a sample, known as ionization, which is destructive. Some concepts are shown in Fig. 2d. Most commonly, X-ray photoelectron spectroscopy (XPS) and Auger electron spectroscopy (AES) are used, but electron energy loss spectroscopy can also be applied to study materials properties at nanoscale spatial resolution. In XPS, electrons are ejected upon excitation from an inner-shell (atom-like) energy level and the energy of this emitted core electron is measured. In contrast, AES measures electrons that are typically ejected from a valence level with a high kinetic energy due to a complex preceding process. In the latter, a core electron (first electron) is ejected from the sample, typically by an external electron beam, then the corresponding vacancy is filled by a (second) electron from an outer shell and finally the afore mentioned (third) Auger electron is emitted from a valence level. As the binding energy of the auger electron is usually much smaller, hence relaxation energy from the other electron is usually large enough. Ejected electrons are characteristic to a system, providing information about elements and binding energies. X-ray spectroscopy is especially powerful for surface analysis^59^ and a recent perspective on X-ray spectroscopy using ML can be found in ref. 60.

### From structure to spectra

**Fig. 4:**
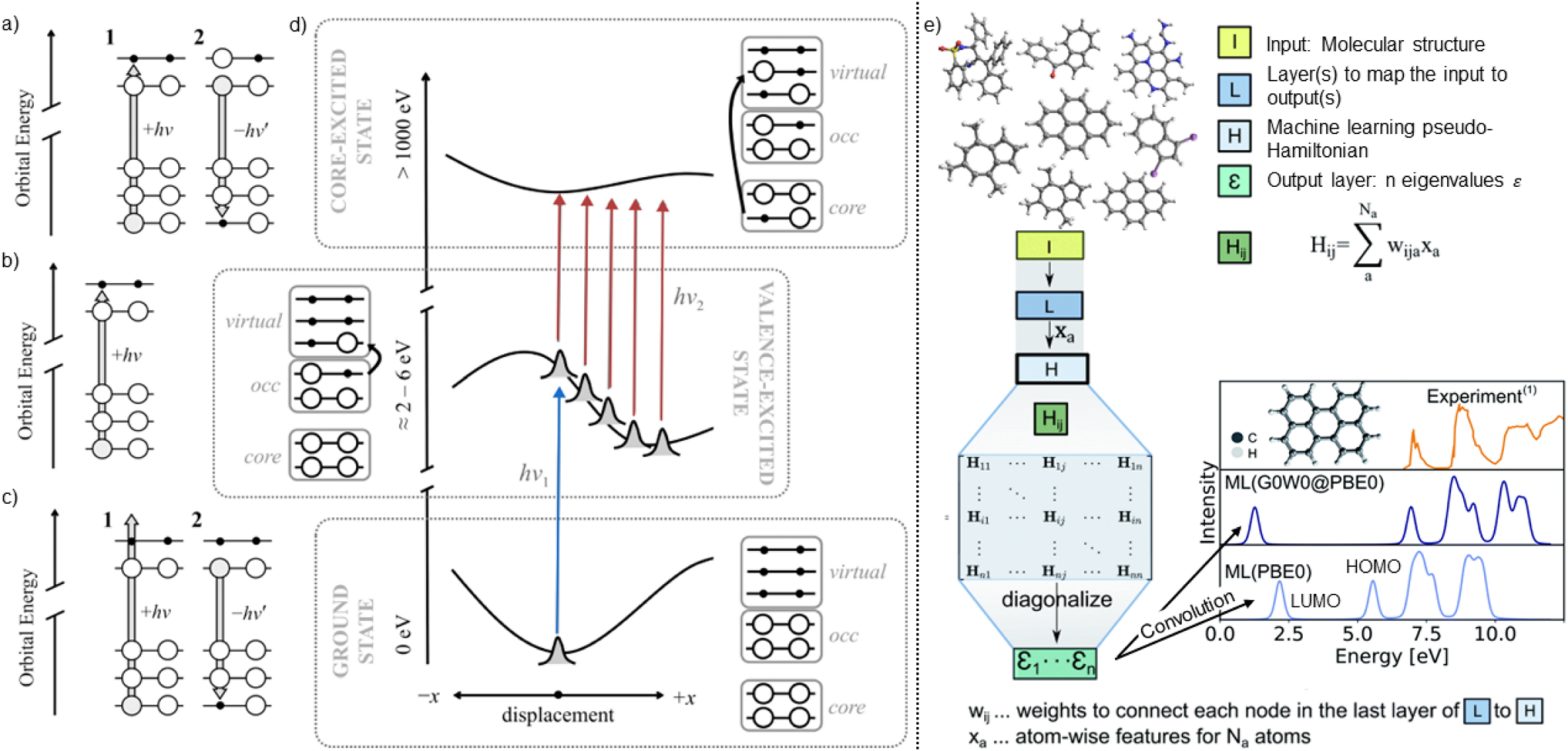
Illustration of (a) XES, (b) XAS, and (c) resonant X-ray emission (RXES) and resonant inelastic X-ray scattering (RIXS). (d) UV/visible-pump-X-ray-probe spectroscopy to follow ultrafast excited-state dynamics. A wavepacket is excited by a pump pulse (blue), where an X-ray probe pulse (red) creates a core-excited state. (e) Learning of multiple electronic states (quasiparticle energies) *via* physically-inspired machine learning. An internal pseudo-Hamiltonian is generated that allows a smooth representation of electronic energies. The quasiparticle energies, *ε*, are then obtained *via* diagonalization of the pseudo-Hamiltonian and can be convoluted (Gaussian broadening) to obtain (inverse) photoemission spectra. Images of (a–d) are adapted from ref. 60 and (e) is adapted from ref. 67 with permission from RSC under CC-BY 3.0.

Pioneering works on the prediction of X-ray absorption spectra have been conducted by Penfold and co-workers. The authors have developed a deep neural network that was trained on about 9000 Fe K-edge X-ray absorption near-edge structure spectra (XANES) obtained from the Materials Project database. XANES are especially powerful as they provide a fingerprint of the electronic and atomic structure around an atom. As inputs to the neural networks, the authors used local information around the Fe absorption site to qualitatively predict peak position and intensities of close-to-equilibrium structures.^61^ In a subsequent study, their approach was extended (XANESNET) to predict X-ray absorption spectra of nine first-row transition metal K-edges by discretizing spectra into 376 equally spaced points and learning peak intensities.^62^ The model has further been applied during molecular dynamics simulations to investigate structural changes due to temperature jumps in a sample,^63^ to predict L_2/3_-edge spectra^64^ and valence-to-core X-ray emission spectra^65^ and the authors have further investigated the effect of descriptors on the performance of neural networks for spectra predictions and found that local descriptors were superior to the global Coulomb matrix.^66^ Different types of excitations in X-ray spectroscopy are illustrated in Fig. 4a–c with UV/visible-pump-X-ray-probe spectroscopy visualized in panel (d). As can be seen, a pump probe excited a valence electron, while a probe X-ray pulse excites core electrons to study time-dependent phenomena.

Aarva *et al.*^68,69^ studied X-ray photoelectron spectra of functionalized amorphous carbonaceous materials with Gaussian Process Regression. For their work, the core-electron binding energies were encoded into a Gaussian kernel, which was combined with a kernel encoding the structures of the material in a linear fashion. Average fingerprint spectra of surfaces of different functionalization could be obtained *via* clustering techniques. These clusters were then used to compute spectra to fit experimental data allowing for semi-quantitative information of the composition of a material.

Another technique worth mentioning in this section is photoelectron spectroscopy, also known as photoemission spectroscopy. This technique makes use of the photoelectric effect and measures emitted electrons for the determination of binding energies of electrons. For ionization, X-ray, X-UV or UV photons can be applied. To characterize unoccupied energy levels, inverse photoemission spectroscopy can be used, where electrons are coupled to unoccupied electronic states. Decay processes can be measured. Due to the low energy of electron beams shot at the sample, *i.e.*, around a few 10 s of eVs, the method is especially sensitive to surface characterization. In Inelastic Photoelectron Spectroscopy (IPES) electrons are used to radiate a surface. It is a technique used in surface science to study the electronic structure of materials by measuring the kinetic energy of electrons emitted. The first ML study applied to photoemission spectroscopy was conducted by Ghosh *et al.*,^70^ who compared different regressor and descriptors, *i.e.*, multi-layer, convolutional, and deep tensor neural networks, in combination with the Coulomb matrix and a message-passing framework, respectively, to predict 16 orbital energies to approximate orbital energies of organic molecules in the QM7 and QM9 data sets. To obtain higher accuracy of photoemission spectra, the GW method could be used, and quasiparticle energies could be learned compared to Kohn–Sham orbital energies. Therefore, Westermayr *et al.*^67^ have combined a physics-inspired model for orbital energies at Kohn–Sham level of theory with a Δ-ML model to correct these values to quasiparticle energies for accurate prediction of (inverse) photoemission spectra, ionization potentials and electron affinities of molecules in the OE62 (ref. 71) data set. The latter concept is visualized in panel (e) of Fig. 4.

### From spectra to (electronic) structure

An interesting approach to describe the electronic properties of transition metal ions was performed by Lüder,^72^ who developed three types of neural networks that take computational or experimental 2p XAS spectra at the L-edge as an input. In their approach, the authors made use of the crystal-field multiplet theory and employ a Hamiltonian, *Ĥ*, to obtain the intensities of an XAS spectrum:
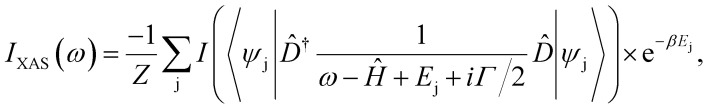
where *E*_j_ is the eigenenergy of state j, *Z* the partition function, *Γ* an imaginary shift, and *D̂* the dipole operator that provides information on the excitation of a p-electron into a d-state. One neural network predicts d-level positions, the second one incorporates information on the influence of temperature (given as the inverse temperature using *β*), and the third network encodes the Hamiltonian, *Ĥ*, in the local atomic orbital basis, such that electronic ground state properties like the occupation number or spin state can be obtained. The method was applied to Co^2+^, Ni^2+^, Fe^2+^, and Mn^2+^ spectra to determine 3d states, core hole lifetimes and Coulomb and exchange interaction screening factors.

Another study performed by Drera *et al.*^73^ computed around 100 000 synthetic X-ray spectra of adventitious carbon-layered materials and trained deep neural networks to detect chemical elements in spectra and quantify them. Labels were the chemical elements, corresponding strongest lines in the spectra and quantitative information. Another attempt to obtain information on the atomic and electronic structure was made by Guda *et al.*,^74^ who learned from Fe:SiO_2_ XANES the relation between spectral properties, such as the peak positions, intensities, or edge positions, and atomistic as well as electronic properties, such as oxidation state, coordination number, bond distances, and bond angles. Recently, a workflow combining ML and experimental obersvables to predict atomistic structures and applied it to oxygen-rich amorphous carbon.^75^

### Data analysis and processing

Most of the ML studies applied to X-ray spectroscopy are in the field of data analysis.^76^ In 2002, Gallagher *et al.* used neural networks to classify experimental X-ray spectra of minerals.^77^ The class of a compound has been learned from the shape of a spectrum by Chatzidakis *et al.* using convolutional neural networks.^78^ Tetef *et al.* applied unsupervised learning to characterize the sulfur bonding environment of sulforganic molecules.^79^ Timoshenko *et al.* used theoretical FXAS to train neural networks on the coordination number of metal nanoparticles.^80^ XAS also served the development of ELSIE by Zhang *et al.*,^81^ which is an Ensemble-Learned Spectra Identification algorithm for spectra analysis.

### Outlook and perspective

As ML models already exist for energies, forces, and dipole moments for a wide range of molecules and materials, we believe that this will soon be the case for orbital energies as well. This can advance X-ray spectroscopy and additionally transfer learning could be applied to generalize across different experimental setups and synchrotron facilities, addressing current limitations in cross-instrument compatibility. Time-resolved ML models will enable real-time analysis of pump-probe experiments and *in operando* studies of catalytic processes, expanding from static structure prediction to dynamic phenomena understanding.

Uncertainty quantification will become crucial for surface analysis applications, where ML models must provide confidence intervals for composition predictions from noisy experimental data. Multi-modal integration combining X-ray techniques with complementary methods (electron microscopy, optical spectroscopy) through joint ML frameworks can provide comprehensive materials characterization.

## Nuclear magnetic resonance (NMR) spectroscopy

Besides optical and X-ray spectroscopy, structure elucidation is possible *via* NMR spectroscopy. NMR uses a magnetic field that interacts with the fractional spin of a nucleus, like ^1^H, ^13^C, ^19^F, or ^31^P, where energy transfer is provided by on-resonance radio frequency pulses at corresponding wavelengths. This concept is shown schematically in Fig. 2e. Measured is the relaxation signal as a response to the excitation, which is then translated to the chemical shift, *δ* = (*ν* − *ν*_0_)/*ν*_0_, *via* Fourier transformation and referencing to the device. In this way, the chemical shifts are universally comparable and is usually expressed in ppm with *ν*_0_ being the chemical shift of a reference compound. The chemical shift depends on the chemical environment of a system, hence information about chemical bonding, electronic structures, and local dynamics can be obtained *via* NMR. Within the gauge-dependent atomic orbital framework, the shielding tensor, *σ*^a^_k*l*_ of atom a:
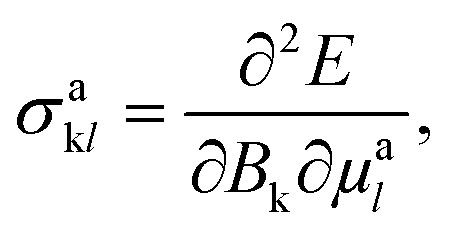
with *B*_k_ being a component of the external magnetic field and μ^a^_*l*_ being the nucleus magnetic moment of the *l*th component. The chemical shifts and scalar coupling constants, *e.g.*, ^1^*J*_CH_, the chemical coupling constant between ^1^H–^13^C, sensitive to the connectivity and especially relevant for three-dimensional structure elucidation.

ML has been applied to NMR spectroscopy relatively late as the modeling of chemical shifts is challenged by a lack of data sets available, their strong dependency on the environment, and the large chemical and combinatorial space possible.^82^ One of the first studies that applied ML to NMR was conducted by Cuny *et al.* in 2016. In their work, the authors used neural networks to predict NMR parameters for crystalline silica polymorphs and silica glasses and ^17^O and ^29^Si quadrupolar couplings.^83^ Similarly, Paruzzo *et al.*^82^ developed SHIFTML, an approach to predict NMR parameters based on DFT for molecular solids and corresponding polymorphs. Their model was further tested on cocaine and a drug-like molecule and was found to be accurate with respect to experimentally measured spectra. Gao *et al.* combined DFT calculations with neural networks to predict ^1^H and ^13^C chemical shifts at close to experimental accuracy.^84^ The authors could further improve the prediction by using a graph convolutional neural network to predict the structural descriptor.^85^

To foster the development of new ML models for NMR spectroscopy, Gupta *et al.*^86^ generated the QM9-NMR data set, an extension of the QM9 data set that contains ^13^C NMR shielding values for C atoms in the QM9 molecules in the gas phase and five different solvents. The authors additionally tested kernel ridge regression and Δ-ML for the prediction of the shielding values. The model gave reasonable accuracy, while noting that additional data for fullerenes or molecules like polycyclic aromatic hydrocarbons might increase the accuracy of Δ-ML for other systems than those present in the QM9 data base. Another study applied Δ-ML to generate CCSD(T)-quality NMR chemical shifts based on DFT.^87^

Besides the prediction of chemical shifts, coupling constants are relevant to determine three-dimensional structures. To accomplish structure discrimination of spectra, Gerrard *et al.*^88^ developed IMPRESSION, a model to predict ^1^H, ^13^C, and ^1^*J*_CH_, couplings of organic molecules. Recently, Cryo-EM, NMR, and ML have been combined to enable the validation of protein dynamics, improving description of allosteric regulations or enzyme catalysis, for instance.^89^ Formula and connectivity of molecular structures could be predicted *via* multi-task ML model that takes two input spectra, *i.e.*, ^1^H-NMR and ^13^C-NMR. The workflow combines a convolutional neural network and a transformer model with the latter being capable of constructing the molecular structure based on many molecular fragments.^90^ Further studies focus on the correlation between surface porosity in nanomaterials and NMR spectra by using multivariate ML that combines partial least squares and dimensionality reduction techniques or quantitative metabolomics and 2D-NMR.^91^

To further advance development on ML for NMR, a Kaggle challenge has been set up to predict 8 different types of scalar couplings of structures in the QM9 data set.^92^ The best 400 models were combined to a meta-ensemble model, which is an ensemble of ensemble methods as some of the best models were ensemble models themselves. By doing so the accuracy could be increased by 7–19 times.

### Outlook and perspective

In the future, we believe that NMR spectroscopy can be advanced by conformational ensemble modeling where ML models predict NMR parameters for dynamic systems, capturing averaging effects. In addition, multi-nuclear prediction models could be developed that use synergistic knowledge of chemical shifts and coupling constants across different isotopes, enabling comprehensive structure elucidation from minimal experimental data. Further, ML can be integrated with cryo-EM and NMR data to validate protein dynamics and allosteric mechanisms, combining structural and dynamic information for comprehensive molecular understanding. Real-time metabolomics applications might emerge through automated and integrated spectral interpretation and biomarker identification *via* ML.

## Mass spectrometry (MS)

Finally, MS has become an indispensable tool to analyze the composition of samples qualitatively and quantitatively and has already made use of ML techniques in the 1960s.^93,94^ MS is often applied in lipidomics, proteomics, and metabolomics that provide information about physical conditions and disease progression in organisms by measuring lipids, proteins, and chemical products of reactions that happen within cells of living organisms as well as their metabolic networks, respectively. MS consists of an ionization source to ionize and fragment a sample, which can then be identified by separating fragments by their mass-to-charge ratio (*m*/*z*). An example of a spectrum is shown in Fig. 2c that can be produced theoretically from molecular dynamics as visualized *via* an arrow from Fig. 2b and c. A mass spectrum is recorded in an ion detection system. When two or more mass analyzers are coupled, the method is referred to tandem MS, MS/MS, or MS^2^. As MS produces massive amounts of data that are highly complex, especially when studying metabolic networks that have not been characterized previously, ML methods are especially promising when being applied to data analysis.^95,96^ A recent review on ML in MS can be found in ref. 97.

### From structure to spectra

In contrast to the previous methods, the generation of synthetic data for MS further relies on the prediction or simulation of reactions to determine the fragments of which spectra should be predicted.^98,99^ ML can thus not only enhance the process of spectra prediction but can also significantly speed up the simulation of fragments. Several tools exist that apply quantum chemistry methods, often in combination with molecular dynamics, to predict fragments that are reviewed in ref. 94. This concept is further shown in Fig. 2b and c, where a dissociative potential is overcome, and fragments are formed. A vast number of methods have been developed by Grimme *et al.* using their semiempirical tight-binding method.^99–102^ This method is versatile and can be employed to almost any system as its semiempirical character allows for computationally efficient simulations also of extended systems. In addition, Grimme and co-workers recently presented a computational workflow, QCxMS2, to compute electron ionization MS spectra by combining Monte-Carlo simulations, automated reaction network, and transition state theory.^103^ One of the first approaches that made use of ML is DENDRAL developed in 1965 to model fragmentation processes. Other tools are MASSIMO by Gasteiger and co-workers,^104^ or VENUS by Hase, Spezia and coworkers.^105^ Collision-induced dissociation fragmentation processes can be modeled using MetISIS^106^ or CFM-ID,^107^ the latter is based on experimental data. NEIMS uses neural networks to predict EI MS spectra and extend the coverage of MS libraries with synthetic data.^108^ An advantage of using ML to simulate fragmentation processes compared to using chemical intuition or predefined rules is the reduction of bias. All of these approaches make a number of approximations and even experimental results differ from one apparatus to the next, such that perfect agreement is seldomly reached.

### From spectra to structure

One of the biggest challenges when trying to identify a structure from MS is the huge chemical space that needs to be considered. Generators can be used to produce all possible compounds that satisfy a certain mass and chemical composition. However, this approach quickly becomes infeasible with increasing system size. For instance, the molecular formular C_8_H_6_N_2_O with a mass of 146 Da comprises more than 100 million possible structures.^93,94^ To re-rank molecular formula candidates ZODIAC^109^ can be used that applies similarity networks and Bayesian statistics for scoring.

Once a set of structures is obtained, different approaches can be used to predict a spectrum, which can then be used to identify an analyte:^93,94^ (1) rule-based fragmentation spectrum prediction, which uses fragmentation rules to predict spectra of candidate molecules and compares them to the query spectrum, (2) combinatorial fragmentation, which fragments all possible candidates and tries to explain peaks in the query spectrum, (3) competitive fragmentation modeling,^110^ which predicts a fragmentation spectrum including peak intensities by estimation of the probability of a fragmentation event using generative models trained on experimental data, and (4) molecular fingerprint prediction,^111^ combining fragmentation trees with kernel ridge regression models for the prediction of molecular fingerprints from MS data, which can then be compared to structure databases.^93^ The competitive fragmentation modeling tool is available at the web server prediction,^111^ Data from CFM-ID generated MS^2^ can be used to train Deep Mass,^112^ a neural network-based approach for scoring similarities of metabolites. Another approach is Sepc2Vec^113^ that computes similarity between MS. In the field of omics, one challenge is related to the missing robustness of ML due to a lack of high-quality, comprehensive, and standardized datasets. Strategies to combat data scarcity, for instance, *via* multitask learning or improved data standardizations could be avenues to advance this field.^114^

### Data analysis

Classification of MS based on molecular fingerprints can be pursued with CANOPUS. This model applies support vector machines to predict molecular fingerprints based on fragmentation spectra and uses neural networks for classification of metabolites.^115^ To assign metabolites, METASPACE-ML has been developed, which improves the METASPACE engine, an false discovery rate-controlled metabolite annotation tool for imaging MS spectrometry.^116,117^

### Outlook and perspective

Especially the field of MS can profit from ML as various universal and foundational models are currently being developed for a wide range of molecules and materials that can predict collision-induced dissociation patterns for novel metabolites and drug metabolites, extending beyond existing spectral libraries. Reinforcement learning could be integrated into the process to optimize fragmentation conditions in real-time and active learning approaches could be applied to search for holes in ML potentials and spectral libraries.

## Challenges and prospects

While ML has significantly improved spectroscopy, there are still several open challenges that need to be overcome that currently restrict high-throughput screening and fast structure and composition identification, especially of complex mixtures or extended systems. One of the main challenges is the combinatorial complexity when dealing with large systems like proteins or lipids, frequently analyzed with MS, makes spectral interpretation challenging. With the advent of versatile ML models that can be applied to almost any types of systems, from organic molecules to hybrid organic–inorganic interfaces,^35,118^ new avenues arise that can also enhance spectroscopy. While these methods are powerful to predict almost any types of systems, enabling high-throughput screening, it comes at the cost of accuracy. Nevertheless, especially in the case where many systems should be screened and compared qualitatively, their accuracy can be considered sufficient. Fine-tuning of foundational models also improves accuracy. As an example, these models could be used to simulate molecular dynamics and generate fragments that could give rise to mass spectra. In addition, their adaptations to not only predict potential energy surfaces but also dipole moments and other properties could significantly enhance the generation of optical spectra in a high-throughput fashion, especially considering excited-state foundational versions.^36,37^ Still, the simulation of molecular dynamics to generate spectra is time consuming even when applying ML. In this regards, new algorithms, for instance, based on reinforcement learning or generative models,^119^ that might allow to directly predict fragments and structures with specific properties could further enhance this field.

Another aspect is that most models are trained on theoretical data, while direct application of ML to experimental data is sparse. How to deal with the comparably noisy and little data obtained from experiments to obtain robust ML models that can further provide information on the uncertainty associated with experimental measurements still needs to be investigated. This step requires high-throughput experimentations that are likely to become available with the automation of processes and miniaturization of technology.^120,121^ In addition, automation and robotics (with well-equipped laboratories that guarantee a constant temperature) could dramatically reduce the noise produced by individuals conducting experiments. Further, when dealing with experimental data, different instruments and setups can lead to varying results, which can make it difficult to develop models that can be easily transferred between different systems. Models developed for one instrument may not be applicable to another instrument without significant modifications. Transfer learning or domain adaptation techniques could help to adapt a model to new instruments or experimental setups.

One area of huge potential is the interpretability of ML models, as they are currently mainly operated as “black box” models without providing insight into the underlying physical processes that generate the data. Allowing for interpretability and explanation in ML models could pave the way towards the discovery of new chemical rules hidden behind the high complexity of data. One way towards achieving this goal is the use of tools of explainable artificial intelligence,^122–125^ or to incorporate domain knowledge by using hybrid models that combine machine learning with physics-based models.

## Author contributions

J. W. and P. M. worked out the concept and wrote the manuscript.

## Conflicts of interest

There are no conflicts to declare.

## Data Availability

No primary research results, software or code have been included and no new data were generated or analysed as part of this review.
